# What the papers say

**DOI:** 10.1093/jhps/hnu017

**Published:** 2014-11-11

*Journal of Hip Preservation Surgery* (*JHPS*) is not the only place where work in the field of hip preservation may be published. Although our aim is to offer the best of the best, we continue to be fascinated by work that finds it way into journals other than our own. There is much to learn from it so *JHPS* has selected six recent and topical articles for those who seek a brief summary of what is taking place in our ever-fascinating world of hip preservation. What you see here are the mildly edited abstracts of the original articles, to give them what *JHPS* hopes is a more readable feel. If you are pushed for time, what follows should take you no more than 10 min to read. So here goes …

## CAN MECHANICAL FACTORS EXPLAIN DEVELOPMENT OF CAM-TYPE DEFORMITY?

It is well known that a cam-type deformity is more prevalent among young active adults and may increase the risk of hip osteoarthritis [1]. Researchers in Delft and Rotterdam, The Netherlands hypothesized that the loading conditions experienced during certain types of vigorous physical activities may stimulate the formation of cam-type deformity. They proposed that the growth plate shape modulates the influence of mechanical factors on the development of cam-type deformity.

The study used finite element models of the proximal femur with an open growth plate to study whether mechanical factors could explain the development of cam-type deformity in adolescents. Four different loading conditions (representing different types of physical activities) and three different levels of growth plate extension towards the femoral neck were considered. Mechanical stimuli at the tissue level were calculated by means of the osteogenic index (OI) for all loading conditions and growth plate shape variations.

It was found that loading conditions and growth plate shape influence the distribution of OI in hips with an open growth plate, thereby driving the development of cam-type deformity. In particular, specific types of loads experienced during physical activities and a larger growth plate extension towards the femoral neck increase the chance of cam-type deformity.

The authors concluded that specific loading patterns seemed to stimulate the development of cam-type deformity by modifying the distribution of the mechanical stimulus. This would be consistent with recent clinical studies and reveals mechanobiological mechanisms that trigger the development of cam-type deformity. Avoiding these loading patterns during skeletal growth might be a potential preventative strategy for future hip OA.

## DOES GENDER DICTATE CLINICAL PRESENTATION AND DISEASE CHARACTERISTICS OF FEMOROACETABULAR IMPINGEMENT?

Cam-type femoroacetabular impingement (FAI) is generally described as being more common in males, with pincer-type FAI being more common in females [2]. The purpose of this prognostic level I study from Washington, USA was to determine the effect of sex on FAI subtype, clinical presentation, radiographic findings and intraoperative findings in patients with symptomatic FAI.

The study compared cohorts of 50 consecutive male and 50 consecutive female patients who were undergoing surgery for symptomatic FAI. Detailed information regarding clinical presentation, radiographic findings and intraoperative pathology was recorded prospectively and analysed. FAI subtype was classified on the basis of clinical diagnosis and radiographic evaluation.

The study found that female patients had significantly greater disability at presentation, as measured with use of the modified Harris hip score, the Western Ontario and McMaster Universities Osteoarthritis Index (WOMAC), the Hip Disability and Osteoarthritis Outcome Score, and the SF-12 (12-Item Short Form Health Survey) physical function subscore (all *P* ≤ 0.02), despite a significantly lower UCLA (University of California at Los Angeles) activity score (*P* = 0.03). Female patients had greater hip motion (flexion and internal rotation and external rotation in 90° of flexion; all *P* ≤ 0.003) and less severe cam-type morphologies (a mean maximum alpha angle of 57.6° compared with 70.8° for males; *P* < 0.001). Males were significantly more likely to have advanced acetabular cartilage lesions (56% of males compared with 24% of females; *P* = 0.001) and larger labral tears with more posterior extension of these abnormalities (*P* < 0.02). Males were more likely than females to have mixed-type FAI and thus a component of pincer-type FAI (combined-type FAI) (62% of males compared with 32% of females; *P* = 0.003).

The authors concluded that there were distinct, sex-dependent disease patterns in patients with symptomatic FAI. Females had more profound symptomatology and milder morphologic abnormalities, while males had a higher activity level, larger morphologic abnormalities, more common combined-type FAI morphologies, and more extensive intra-articular disease.

## AN ANATOMICAL STUDY OF THE ACETABULUM WITH CLINICAL APPLICATIONS TO HIP ARTHROSCOPY

The clock face has been employed to define the position of labral pathology in relation to identifiable arthroscopically relevant acetabular landmarks [3]. The purpose of this cadaver study, performed in the Steadman Philippon Research Institute Vail, USA, was to qualitatively and quantitatively describe arthroscopically relevant anatomy of the acetabulum. The authors aimed to present a surgical landmark that is located in close proximity to the usual location of labral pathology as an alternative to the midpoint of the transverse acetabular ligament as a reference point.

Fourteen fresh-frozen cadaver hemipelves were dissected to evaluate osseous landmarks and relevant surrounding soft-tissue structures of the acetabulum. With use of a coordinate-measuring device, the researchers determined the location, orientation and relationship of key arthroscopic landmarks and the footprint areas formed by the insertions of the rectus femoris, capsule and labrum.

An analysis of variability of reference points around the acetabulum in relation to the anterior inferior iliac spine (AIIS) revealed that the superior margin of the anterior labral sulcus (psoas-u) was the most consistent anatomic landmark. The AIIS comprised superior and inferior facets, demarcated by the origins of the direct head of the rectus femoris and the iliocapsularis. The inferolateral corner of the footprint of the direct head of the rectus femoris was located 19.2 mm from the acetabular rim and the inferolateral aspect of the iliocapsularis footprint, 12.5 mm from the rim.

The authors concluded that the superior margin of the anterior labral sulcus (psoas-u) was a reliable landmark for reference of the clock face on the acetabulum. They proposed that this point, denoting 3:00, be adopted as the new standard clock-face reference for intra-articular hip structures because of its universal presence and reliable arthroscopic visualization. This marker is also beneficial because of its proximity to the typical location of labral pathology. The data presented provide a comprehensive analysis of pertinent arthroscopically relevant acetabular anatomy. The clinical relevance of the study would be in the establishment of a new standard reference point within the acetabulum to enhance the consistency of interpretation of the location of labral pathology and improve arthroscopic orientation and navigation.

## TREATMENT OF FULL THICKNESS CARTILAGE DEFECTS OF THE HIP WITH AUTOLOGOUS CHONDROCYTE TRANSPLANTATION

Treatment of full-thickness cartilage defects found during hip arthroscopy is a difficult problem [4]. Autologous matrix-induced three-dimensional chondrocyte transplantation using 3D spheroids (ACT 3D) may be an option for treatment. The aim of the study performed in Essen, Germany was to describe the feasibility and first clinical results of ACT 3D with spheroids at the hip.

In this prospective cohort level III study, the authors described their surgical technique and the outcome of 16 patients with chondral defects induced by cam-type FAI. All patients underwent physical examination before the first surgery and again before the second (about 6 weeks later). Further examinations were performed 6 weeks after the second surgery and at an average follow-up period of 16.09 months. At every visit, the non-arthritic hip score (NAHS) and WOMAC were obtained. In addition, patient satisfaction was evaluated during the last follow-up examination by means of a questionnaire.

The NAHS and WOMAC scores had significantly improved 6 weeks after arthroscopic treatment of the cam-type FAI, and a further significant enhancement was seen 6 weeks after the second surgery with application of the chondrocyte spheroids. In the last follow-up, the mean results were equally as good as the second follow-up examination 12 weeks after surgery.

The authors concluded that ACT 3D using spheroids is a feasible method that can be easily performed during arthroscopy. They found the results encouraging and identified the need for further studies to get an impression of the quality grade of this method in comparison with other treatment options in case of chondral defects in the hip.

## HIP MORPHOLOGY WILL INFLUENCE THE PATTERN OF ARTICULAR CARTILAGE DAMAGE

One of the dilemmas in hip preserving surgery is understanding the pattern of injuries cause by various pathologies as definitive treatment of the primary pathology is imperative to prevent further damage and allow healing [5]. In an interesting prospective cohort study (level III) performed in Sapporo, Japan, Kaya *et al.* [5] aimed to the obtain data on chondral damage and compare the damage patterns of various hip disorders.

The data were collected at 100 consecutive arthroscopies, and chondral lesions were recorded on anatomic articular maps divided into different anatomical zones. This geographic zone method made it possible to analyse the International Cartilage Repair Society grade and location in relation to the hip morphology. The distribution and degree of the chondral defects showed a hip morphology-specific pattern. On the acetabular side, there were high incidences of full-thickness defects in the anterior–superior zone and the middle superior zone in patients with FAI (zone 2: 25.4% grade 3, 35.5% grade 4; zone 3: 20.3% grade 3, 37.2% grade 4) and borderline dysplasia (zone 2: 31.2% grade 3, 12.5% grade 4; zone 3: 18.7% grade 3, 25% grade 4). However, in patients with joint laxity, partial-thickness defects were dominant (zone 2: 50% grade 1, 15% grade 2; zone 3: 40% grade 1). In patients with acetabular dysplasia, full-thickness defects extended even to the posterior superior zone (zone 4: 80% grade 4). On the femoral head side, the incidence of full-thickness cartilage injuries was high in patients with FAI and borderline dysplasia compared with those with joint laxity and acetabular dysplasia.

The authors concluded that evaluation of chondral damage using the geographic zone method showed that the pattern of cartilage damage was influenced by hip morphology. This study will help understanding of hip disorder-specific chondral damage patterns and may lead to establishment of appropriate treatment guidelines depending on the primary pathology.

## DOES PRESERVATION OF THE RECTUS FEMORIS ORIGIN DURING PERIACETABULAR OSTEOTOMY COMPROMISE ACETABULAR REORIENTATION?

The original technique of the Bernese periacetabular osteotomy (PAO) has evolved and it has been proposed that sparing the rectus femoris and discontinuing routine arthrotomy may accelerate early postoperative recovery compared with the standard approach [6]. Researchers from Salt Lake City, USA set up a case control study (Level III evidence) to question whether a modified approach for PAO (i) lead to improved pain control immediately after surgery; (ii) lead to improved ambulation during the hospital stay; (iii) lead to shorter stays, less blood loss and shorter surgical times and (iv) compromise acetabular correction?

They retrospectively reviewed 75 patients who underwent PAO for developmental dysplasia of the hip between August 2009 and May 2013. The control group included 44 consecutive patients who underwent a standard Bernese PAO with rectus takedown (RT). The study group consisted of 31 consecutive patients who underwent PAO using a modified rectus-sparing (RS) approach without routine arthrotomy. The groups were similar in age, body mass index and American Society of Anesthesiologists score, but the RT group was comprised of a greater percentage of men than the RS group. Outcome variables were collected from patient charts and included inpatient pain, inpatient ambulation as well as length of stay, estimated blood loss, surgical time and postoperative radiographic measurements. Cohen's *f*^2^ was used to calculate the effect size in the regression analysis and effects were considered small for values <0.15, moderate for 0.15–0.34 and large for values >0.35.

Patients who underwent PAO with a RS approach had less overall pain (RT median 4 versus RS median 2); however, the difference may not have been perceptible to the typical patient (*P* = 0.001, *f*^2 ^= 0.059). Patients treated with the RS approach ambulated similar distances during the hospital stay with a median of 11 feet for the RT group and a median 30 feet for the RS group (*P* = 0.215, *f*^2 ^= 0 .095). Patients in the RT group had a median length of stay of 4 days compared with a median 3 days in the RS group (*P* < 0.001). The median estimated blood loss was greater (*P* = 0.010) in the RT group (median, 500 ml) versus the RS group (median, 300 ml). The median surgical time was longer (*P* < 0.001) in patients undergoing PAO with the RT approach (median, 159.5 min) compared with the RS approach (median, 103 min). Acetabular reorientation based on postoperative radiographs was not compromised by the modified approach.

The authors concluded that the modified approach was straightforward to implement in all patients and did not compromise acetabular fragment mobilization or final positioning. Two of the three key variables that the approach might have influenced (pain and length of stay) were below the minimum clinically important difference and different by only a fraction of a day, respectively. The difference in ambulation was of only modest clinical importance. They did identify the need for more definitive evidence for clinical superiority in terms of pain, ambulation and return of muscle function and proposed the use of more sophisticated instruments such as gait analysis, muscle strength testing and longer-term outcome studies with sensitive instruments.
